# Editorial: Brain-Mind-Body Practice and Health

**DOI:** 10.3389/fpsyg.2017.01886

**Published:** 2017-10-25

**Authors:** Gao-Xia Wei, Gangyan Si, Yi-Yuan Tang

**Affiliations:** ^1^Key Laboratory of Behavioral Science, Institute of Psychology, Chinese Academy of Sciences, Beijing, China; ^2^Key Laboratory of Mental Health, Institute of Psychology, Chinese Academy of Sciences, Beijing, China; ^3^Sport Psychology Center, Hong Kong Sports Institute, Hong Kong, China; ^4^Department of Psychological Sciences, Texas Tech University, Lubbock, TX, United States; ^5^Center for Advanced Study in the Behavioral Sciences, Stanford University, Stanford, CA, United States

**Keywords:** exercise, brain mechanism, mental health, Tai Chi Chuan, breathing, mind-body

Currently, increasing number of human studies emerged to demonstrate the association between “brain-mind-body practice and health” (Lustig et al., 2009; Bezzola et al., 2011; Wei et al., 2013, 2014), which is of great implication for understanding basic scientific issue “mind and body” and providing efficient strategy for clinical practice and health promotion (Lan et al., 2013; Renoir et al., 2013; Acevedo et al., 2016; Muehsam et al., 2017; Tang, 2017). Here in this research topic, we addressed recent findings from theoretical as well as experimental perspective including contributions under the following three headings: (1) intervention studies to investigate the positive effect of brain-mind-body practice on cognition and relevant brain mechanism. The intervention pattern consisted of short-term practice ranging from few hours to several weeks; (2) cross-sectional studies using expert-novice paradigm to explore the behavioral and neural system change induced by extensive brain-mind-body practice; (3) the mediators influence the relationship between practice and health outcomes and (4) new viewpoints on brain-mind-body practice from theoretical perspectives. Here we briefly highlight these articles aiming to deepen our understanding of relevant development in this topic and establish organic connections among these studies as well as the connections between this topic and broader research field. In addition, it offers an academic insight from theoretical and methodological perspectives for readers. To provide a thorough understanding of these contributions, we classified these publications based on study design.

It is challenging to clarify the influence of brain-mind-boy practice on health outcomes because it was misunderstanding that some outcomes could be detectable only after long-term training or practice, which usually takes much time to conduct relevant longitudinal studies. Therefore, researchers found a good solution using acute exercise paradigm only lasting for about 30 min. In this topic, acute practice among healthy and clinical population were also involved. In this topic, Hung et al. employed acute exercise with moderate intensity to investigate its effect on task switching in children with attention-deficit/hyperactivity disorder (ADHD), which indicated that following exercise these children with ADHD exhibited decreased reaction time and increase P3 amplitude compared to control session. This result suggested that single bouts of moderate intensity aerobic exercise might have positive effects on the working memory of children with ADHD. Chen et al. used functional MRI to examine the neural mechanism underlying the effect of acute aerobic exercise on executive function in healthy children. The results also supported the positive effect of aerobic exercise even it only lasted for only 30 min. Moreover, this study found greater activation in brain regions relevant to working memory after exercise compared to resting condition. It is a remarkable attempt to adopt functional MRI technology to explore such effect in spite of the small sample size. Functional MRI was used in another study on motor fatigue (Hou et al.). This study observed the change of brain activation induced by a hand movement task between following an exhaustive exercise. It consistently observed significantly decreased activation in subcortical areas (basal ganglia) during motor task and implied the key role of subcortical areas in motor fatigue by disturbing motor control processing. Additionally, traditional Electroencephalogram (EEG) study was also included in this topic. Henz and Schollhorn explored the difference of brain activity by comparing after vs. before practice among Qigong practitioners and found both physical practice and mental practice had similar increased alpha activity, indicating mental practice is also an efficient way to reach the relaxed state.

Another solution to detect the effect of practice on health is adopting cross-sectional study to compare the differences in behavioral and neural level between experienced practitioners and controls. Wei et al. used functional MRI to investigate the neural correlates underlying the effect of extensive mind-body practice on cognitive control in large-scaled brain network perspective. They recruited Tai Chi Chuan (TCC) practitioners for 10 years of experience as expert group and controls as novice group to scan their brain function during resting state. The results showed that compared to control group, TCC group had significantly decreased fractional Amplitude of Low Frequency Fluctuations (fALFF) in bilateral frontoparietal network (FPN), which was found to be associated with TCC experience as well as cognitive control performance. This study highlights the functionally plastic role of the frontoparietal network in the context of the “immune system” of mental health recently developed in relation to flexible hub theory. Professional athlete is a typical human model to detect the influence of long-term practice on cognition. Here Xu et al. and her colleagues also used functional MRI to scan task-evoked brain activation during action anticipation task. Greater activation in medial frontal cortex was observed in badminton players relative to control group, which addressed the crucial role of medial frontal cortex in perception anticipation. As mentioned above, the consistent findings in prefrontal cortex possibly supported improved top-down regulation benefited from extensive practice, which might be meaningful for treatment of mental disorders. The limitation of this paradigm is that it is not clear if the differences of behaviors or brain between these groups are induced by nature or nurture (practice) (Shors et al., 2014). It is possible that some practitioners have featured brain tissue sensitive to training and they undoubtedly presented better performance in behaviors. However, these original studies offer more insights for further investigations in potential change of behaviors and neural circuits induced by long-term practice.

In view of this limitation, researchers in the field of brain-mind-body practice utilized short-term intervention period for about several weeks to examine the effect of practice in order to exclude the influence of nature or heredity. Baduanjin, a form of Qigong, was employed as an intervention tool to investigate behavioral change including mood and executive control induced by it. The intervention protocol lasted for 8 weeks. As predicted, Chen et al. detected significant improved mood state and executive function. Moreover, an increase in oxygenated hemoglobin in the left prefrontal cortex was observed during the Incongruent Trails test only after exercise intervention. Similarly, Ma et al. observed that 8-week diaphragmatic breathing without any explicit movement was observed to improve cognitive function and negative mood as well as decreased stress level among healthy adults. A combined cognitive training consisting of memory strategy and executive function was examined in this topic. The results demonstrated the effects of cognitive training on both intention-based and stimulus-based actions, which supported the role of mental training on action operation (Niu et al.). Intriguingly, Luo et al. investigated the effects of working memory capacity (WMC) and state anxiety (SA) on attentional control, which supported working memory training benefited to improve attentional control. And the relation between state anxiety and attentional control was also discussed in this paper.

Although most evidences supported the association between exercise and health-related outcomes, the variables mediating such relationship still remains largely unknown. Relevant questions were discussed in this heading. Cardiovascular fitness level, regarded as an important mediator, was confirmed to associate with cognitive performance, which were involved in two separate studies. Song et al. mainly demonstrated how obesity and cardiovascular fitness are associated with the inhibition aspect of executive function from behavioral and electrophysiological perspectives. What makes that all the more remarkable is adopting randomized control observation design to examine the cognitive difference and simultaneously recorded participants' brain activity during operating Stroop task. The results confirmed the hypothesis that the status of being both normal weight and having high cardiovascular fitness is associated with better behavioral and later stages of electrophysiological indices of cognitive function. Regarding the cognitive component benefited from cardiovascular fitness, Chu et al. used event-related desynchronization (ERD) and event-related synchronization (ERS) to explore group difference of the same cognitive task. This findings finally suggested that such cognitive advantage is related to the inhibition of task-irrelevant information and those processes required the devotion of greater amounts of attentional resources to a given task. Moreover, trait self-control is another factor to influence the relationship between motivation toward exercise and subjective wellbeing by using structural equation modeling (Briki), which attached importance for motivation during exercise and provided important insight for effective exercise instruction.

Theoretical approach on the relationship between brain-mind-body practice and health is also addressed in this topic. Shen et al. elaborated the theoretical framework of triadic interaction of brain-mind-body (TIBMB) for creative insight. In the opinion, it is emphasized that the brain is separated from the body, which can benefit identifying the role of body components in different psychobiological/biopsychological activities and also manifest the zeitgeist of the embodied approach. By contrast, Tang et al. suggested in his opinion that mind and body have to be integrated to explain the effect of mindfulness practice because mindfulness meditation includes three components that interact closely to constitute a process of enhanced self-regulation: enhanced attention control, improved emotion regulation and altered self-awareness. Mind-body practice is a holistic system and works through the integration of different ingredients rather than separated components to achieve the beneficial effects. Moreover, an interesting approach in mind-body-brain practice is optimal performance. Regarding the association between body and brain, Cheron (2016) put forward a new neuroscience perspective to investigate one form of optimal experience—“flow” sate, which pointed out the possible way to measure psychological “flow” with EMG and EEG technology. Similarly, another form of optimal experience—clutch state was attached great importance in investigating mind-body-brain association (Swann et al., 2017). In this issue, it is firstly demonstrated the relationship among optimal performance and mindfulness training (Tang and Bruya). Also the necessity is addressed to explore underlying mechanisms (e.g., key biomarkers) of mind-body interaction and optimal performance.

In summary, these contributions in this research topic cover majority forms of brain-mind-body practice including mindfulness, Tai Chi Chuan, Qigong, cognitive training and aerobic exercise. Readers who are interested in any brain-mind-body practice could have access to basic knowledge on theoretical background and get a glimpse of new development and research findings. The publications in this special issue involve multi-modal techniques to uncover the effect of practice in psychological, physiological, neurobiological, and immunological levels. Thus, all these studies enrich our understanding of neural mechanisms underlying healthy behaviors as well as the association between mind, body and brain, and offer new insights for developing possible behavioral practice interventions in subjects with neurological or mental dysfunction.

## Author contributions

GW drafted and revised the manuscript. GS and YT finalized and revised the manuscript.

### Conflict of interest statement

The authors declare that the research was conducted in the absence of any commercial or financial relationships that could be construed as a potential conflict of interest.
